# Irradiation-Induced Defects and Functional Regulation in Metallic Materials: A Review

**DOI:** 10.3390/nano16211372

**Published:** 2026-10-27

**Authors:** Guang Hu, Tingting Zhou, Jing Tang, Kuankuan Liu, Ge Zhao, Junqi Wang

**Affiliations:** 1 School of Nuclear Science and Technology, Xi’an Jiaotong University, Xi’an 710049, China; guanghu@xjtu.edu.cn; 2 School of Human Settlements and Civil Engineering, Xi’an Jiaotong University, Xi’an 710049, China; 372498563@stu.xjtu.edu.cn (T.Z.); j.tang@stu.xjtu.edu.cn (J.T.); lk20010422@stu.xjtu.edu.cn (K.L.); 2234123164@stu.xjtu.edu.cn (G.Z.)

**Keywords:** metallic materials, irradiation defects, radiation damage, defect engineering, functional materials, surface engineering

## Abstract

Irradiation has long been regarded as a major source of degradation in metallic materials because energetic particles continuously generate irradiation-induced defects that lead to hardening, embrittlement, swelling and creep. Beyond its detrimental effects, irradiation has long been employed to modify material microstructures and properties. More recently, increasing attention has been directed toward understanding and deliberately controlling irradiation-induced defect evolution to regulate material properties within a broader defect-engineering framework. This review systematically summarizes the fundamental damage mechanisms induced by irradiation in metallic materials, with particular emphasis on irradiation hardening, embrittlement, swelling and creep. Subsequently, advances in the functional utilization of irradiation-induced defects are reviewed from three perspectives: structural modification and mechanical property enhancement, irradiation-regulated magnetic properties, and surface and interface functionalization. Representative studies demonstrate that irradiation can effectively induce nanocrystallization, refine microstructures, regulate magnetic anisotropy and Curie temperature, and improve corrosion resistance, wear resistance and biocompatibility through controllable defect engineering. Finally, the remaining challenges associated with defect controllability, quantitative structure–property relationships and engineering implementation are discussed, together with future opportunities enabled by advanced characterization, multiscale modeling and intelligent materials design. This review highlights the dual role of irradiation as both a source of material degradation and a versatile tool for microstructural and functional regulation, providing useful guidance for the development of next-generation structural and functional metallic materials for applications in nuclear energy, aerospace, biomedical engineering and advanced manufacturing.

## 1. Introduction

Metallic materials employed in nuclear fission reactors, fusion devices, aerospace systems, particle accelerators, and other advanced nuclear technologies are continuously exposed to harsh irradiation environments, where they are bombarded by high-energy particles such as neutrons, ions, electrons, protons, and γ-rays. The structural integrity and irradiation tolerance of these materials are critical to the safety, reliability, and service lifetime of engineering systems. Unlike conventional environmental factors such as elevated temperature, mechanical stress, and corrosion, irradiation induces direct energy deposition into the crystal lattice, triggering atomic displacement events and generating large populations of nonequilibrium defects. The accumulation and evolution of these irradiation-induced defects continuously alter the microstructure of metallic materials, eventually leading to the degradation of their macroscopic properties [1,2]. Therefore, understanding the damage mechanisms and microstructural evolution of metallic materials under irradiation has become a fundamental scientific issue for the development of next-generation radiation-resistant structural materials and is essential for the advancement of nuclear energy systems, fusion reactors, and deep-space exploration technologies.

Beyond its well-established role as a source of material degradation, irradiation has also long been employed to modify material microstructures and properties. Meanwhile, advances in irradiation science have increasingly enabled the formation and evolution of irradiation-induced defects to be understood and regulated from a defect-engineering perspective. It has been demonstrated that high-density grain boundaries, heterogeneous interfaces, and nanoscale precipitates can serve as efficient defect sinks, promoting the recombination and annihilation of vacancies and self-interstitial atoms (SIAs), thereby suppressing the formation of voids and dislocation loops and significantly improving irradiation resistance. For example, Liu et al. [3] systematically summarized the irradiation response of nanocrystalline metals and demonstrated that grain boundaries effectively absorb irradiation-induced point defects, leading to the formation of void-denuded zones in Cu, Ni, and stainless steels, which substantially suppress irradiation swelling and microstructural degradation. Interface engineering has also been extensively investigated as an effective strategy for enhancing irradiation resistance by regulating grain-boundary characteristics, heterophase interfaces, and nanoscale layered structures to improve defect absorption and annihilation. These developments demonstrate that a detailed understanding of irradiation-induced defect behavior not only provides the basis for mitigating radiation damage but also offers opportunities for the deliberate regulation of material microstructures and properties.

Metallic materials are widely used as structural materials in nuclear energy systems and other irradiation environments, and their irradiation response has been extensively investigated since the mid-twentieth century, with early studies laying the foundations for the understanding of radiation damage in metals [4,5,6]. Significant progress has been achieved in a wide range of metallic systems, including austenitic stainless steels, ferritic/martensitic steels, nickel-based alloys, titanium alloys, tungsten, and high-entropy alloys [7,8,9,10,11]. Research has expanded to encompass the formation and evolution of irradiation-induced defects, interface-mediated irradiation responses, multiscale modeling, and irradiation damage regulation. Meanwhile, advanced characterization techniques, such as transmission electron microscopy (TEM), atom probe tomography (APT), synchrotron radiation, and neutron scattering, together with computational approaches such as first-principles calculations, have greatly deepened the understanding of irradiation-induced microstructural evolution. Furthermore, the integration of machine learning and multiscale simulations has opened new avenues for the accelerated design and optimization of radiation-resistant metallic materials.

Despite considerable progress in radiation materials science, existing reviews have provided important but often topic-specific perspectives on irradiation effects in metallic materials. For example, previous reviews have examined theoretical models of irradiation hardening and embrittlement [12], irradiation-based regulation of magnetic properties [13], and electron-beam-based surface modification [14,15]. These studies have advanced the understanding of individual damage mechanisms and irradiation-based modification strategies. However, a broader synthesis connecting irradiation-induced defect evolution with both material degradation and the deliberate utilization of irradiation for functional regulation remains valuable. To identify relevant literature, searches were conducted in the Web of Science database using combinations of keywords related to irradiation-induced defects, radiation damage mechanisms, and irradiation-enabled property regulation. Representative search terms included irradiation-induced defects, irradiation hardening, irradiation embrittlement, irradiation swelling, irradiation creep, magnetic properties, and surface modification. Publications were selected based on their relevance to irradiation effects in metallic materials and their contribution to understanding defect evolution, damage mechanisms, or irradiation-enabled functional applications. Earlier seminal studies were also included where necessary to establish fundamental concepts and mechanisms.

Building on this literature base, this review provides a comprehensive overview of irradiation-induced defects in metallic materials, focusing primarily on research advances over the past two decades while integrating defect formation and evolution, damage mechanisms, and functional utilization within a common defect-centered framework. Particular emphasis is placed on irradiation-induced hardening, embrittlement, swelling, and creep, as well as the functional utilization of irradiation for structural modification and mechanical-property enhancement, magnetic-property regulation, and surface/interface functionalization. By linking defect generation, migration, clustering, interactions with dislocations and interfaces, and annihilation to the resulting microstructural evolution and property changes, this review examines how irradiation-induced defects can contribute to material degradation or be exploited for targeted property regulation. For surface and interface functionalization, this review also considers irradiation-associated processes, including solute redistribution, phase evolution, and rapid melting and solidification. Finally, the challenges associated with defect controllability, quantitative structure–property relationships, and engineering implementation are discussed. By integrating these complementary aspects of irradiation research, this review aims to provide mechanistic insights into the dual role of irradiation as both a source of material degradation and a tool for functional regulation, thereby supporting the development of next-generation radiation-tolerant and functionally tailored metallic materials.

## 2. Irradiation Damage Mechanisms in Metallic Materials

Metallic materials are the most widely used structural materials in nuclear reactors, fusion devices, aerospace systems, and particle accelerators, where their service performance directly determines the safety and reliability of engineering components. Under irradiation, metals may experience a variety of degradation phenomena, including irradiation hardening, embrittlement, swelling, irradiation creep, and phase instability [16]. Meanwhile, irradiation-induced microstructural evolution can also be deliberately exploited to tailor material properties, making metallic materials an important platform for both irradiation damage research and functional material design. Among various metallic systems, face-centered cubic (FCC) and body-centered cubic (BCC) alloys have been the most extensively investigated owing to their broad engineering applications [17]. FCC metals, such as Cu, Ni, and austenitic stainless steels, possess densely packed crystal structures and excellent ductility, allowing irradiation-induced point defects to migrate readily and evolve into dislocation loops, voids, and nanoscale precipitates. In contrast, BCC metals, including Fe, Cr, and Fe-Cr alloys, exhibit superior high-temperature stability and are widely employed in fusion and fast reactor systems. Their irradiation response is primarily characterized by the formation of dislocation loops, α precipitation, and irradiation hardening. Hexagonal close-packed (HCP) metals, including Zr, Mg, and Zn, exhibit irradiation responses that are strongly influenced by their crystallographic anisotropy and deformation characteristics. Among these materials, Zr and its alloys are particularly relevant to nuclear applications and have been extensively investigated under irradiation. Representative metallic systems studied to date include Fe-based alloys (e.g., reduced activation ferritic/martensitic steels, FeCrAl alloys, and stainless steels), Cu-based alloys, Ni-based alloys, Al-based alloys, Ti-based alloys, and Cr-Zr protective coatings. Although these materials exhibit distinct irradiation responses owing to differences in crystal structure and alloy chemistry, their microstructural evolution fundamentally originates from the generation, migration, and interaction of irradiation-induced defects with dislocations.

Nevertheless, although neutron, ion, proton, and electron irradiation can all generate displacement damage, they do not necessarily produce equivalent defect populations or spatial distributions because their projectile–target interactions and subsequent defect evolution differ. Owing to their high penetration, neutrons can produce damage throughout a comparatively large specimen volume. Neutron-induced recoil atoms generate displacement cascades, and nuclear reactions may also produce transmutation gases such as helium and hydrogen, depending on the neutron spectrum and alloy composition [18,19]. Accelerator-based heavy-ion irradiation can achieve high and controllable displacement damage rates, but conventional ion energies generally confine damage to a finite depth. The resulting depth-dependent damage profile, implanted-ion effects, high damage rates, and nearby free surfaces may influence defect evolution and complicate comparisons with neutron-irradiated materials [20]. Proton irradiation also produces displacement damage through interactions with target atoms, but the penetration depth and damage profile depend strongly on the incident energy and material. Under appropriately selected conditions, proton irradiation can provide a deeper damage region than conventional heavy-ion irradiation; however, implanted hydrogen and depth-dependent damage can affect the interpretation of experimental results [21]. For electron irradiation of metals under commonly used conditions, the resulting defects are generally more spatially dispersed, with less pronounced dense displacement cascades than those typically associated with energetic neutron and heavy-ion irradiation. Electron irradiation is therefore useful for investigating point-defect properties and kinetics, but it does not necessarily reproduce cascade-dominated damage. Consequently, displacement damage expressed in displacements per atom (dpa) provides a useful basis for comparing irradiation conditions, but similar dpa values do not guarantee equivalent retained defect populations or subsequent microstructural evolution. Comparisons among irradiation studies should therefore consider irradiation species and energy, damage rate, temperature, damage-depth distribution, transmutation-gas production, implanted species, and initial microstructure rather than relying on dpa alone. These differences provide important context for interpreting the irradiation-induced hardening, embrittlement, swelling, and creep discussed in the following sections.

### 2.1. Irradiation Hardening

Irradiation hardening is one of the earliest and most common irradiation-induced phenomena in metallic materials. Its fundamental mechanism lies in the obstruction of dislocation motion by irradiation-induced defects. When energetic neutrons, ions, or electrons bombard a metallic lattice, elastic collisions transfer kinetic energy to lattice atoms, producing primary knock-on atoms (PKAs). These energetic PKAs subsequently initiate displacement cascades, generating large numbers of Frenkel pairs consisting of vacancies and SIAs within an extremely short timescale [22]. Under thermal activation, these point defects migrate and aggregate into defect clusters, including dislocation loops, voids, gas bubbles, and nanoscale precipitates. As irradiation proceeds, the increasing defect density provides progressively stronger obstacles to dislocation motion, thereby increasing the critical resolved shear stress required for plastic deformation [23,24]. Consequently, the yield strength and hardness of the material increase, giving rise to irradiation hardening.

**Figure 1:**
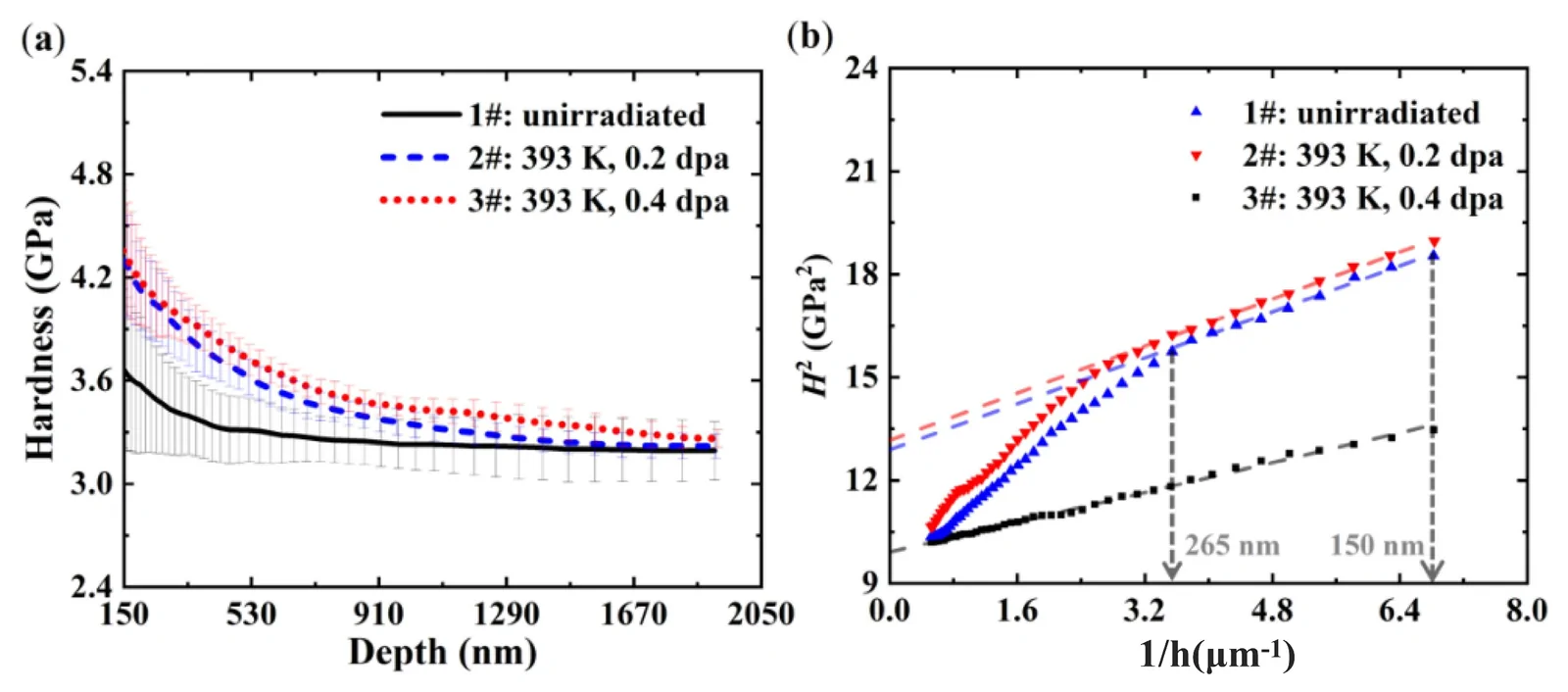
(**a**) Hardness–depth curves and (**b**) H^2^–1/h plots of CLAM steel after 6 MeV Si^3+^ ion irradiation, where H denotes hardness and h denotes indentation depth. In (**b**), the inclined dashed lines represent linear fits based on the Nix–Gao model, while the vertical dashed lines indicate indentation depths of 150 and 265 nm, corresponding to the lower limit for hardness analysis and the approximate onset of substrate effects, respectively. Reprinted from Ref. [25].

The extent of irradiation hardening is governed not only by irradiation dose but also by dose rate, irradiation species, particle energy, temperature, and the intrinsic microstructure of the material. These factors collectively influence the production, survival, migration, and clustering of irradiation-induced defects, thereby determining the number, size, and distribution of obstacles to dislocation motion. The dominant strengthening obstacles vary with the material system and irradiation conditions. In FCC alloys, dislocation loops and voids are generally the dominant strengthening obstacles, whereas irradiation-induced α precipitates, dislocation loops, and nanoclusters collectively contribute to hardening in BCC Fe-Cr alloys. In nanocrystalline metals and heterogeneous materials, abundant grain boundaries and heterophase interfaces serve as efficient defect sinks, promoting vacancy-interstitial recombination and suppressing excessive defect accumulation. Consequently, grain boundary engineering, interface engineering, and precipitate regulation have become effective strategies for mitigating irradiation hardening while improving irradiation tolerance. To verify the underlying hardening mechanism, extensive experimental investigations have been carried out in various metallic systems. For example, Liu et al. [25] investigated irradiation hardening behavior in Chinese low activation martensitic (CLAM) steel by combining Si-ion irradiation and nanoindentation measurements. After irradiation with 6 MeV Si^3+^ ions at different doses, the hardness of CLAM steel increased compared with the unirradiated condition, and the degree of irradiation hardening increased with increasing irradiation dose. The extrapolated bulk hardness increased from 3.15 GPa in the unirradiated sample to 3.59 and 3.63 GPa after irradiation at average doses of 0.2 and 0.4 dpa, respectively. This hardening behavior was attributed to irradiation-induced defects, such as dislocation loops, which act as obstacles to dislocation glide. The dose-dependent hardening trend was further described by the dispersed barrier hardening model, suggesting that the increase in defect density and size contributes to the enhanced resistance against dislocation motion (Figure 1).

Dose-rate effects have also been investigated in reactor pressure vessel (RPV) steels. Fujii et al. [26] reported greater hardening at lower dose rates in A533B steel containing 0.09 wt.% Cu under Fe-ion irradiation at 290 °C. Their microstructural analyses showed that the dose-rate dependence of hardening was primarily associated with the evolution of solute atom clusters. This finding indicates that the relationship between irradiation exposure and hardening depends not only on accumulated dose but also on dose rate and the resulting microstructural evolution.

In addition to Fe- and Cr-based alloys, irradiation hardening has also been extensively investigated in Cu-based alloys. Iwase et al. [27] investigated the effects of 1.5 MeV electron irradiation on Cu-4.2 at.% Ti alloys and found that both the Vickers hardness and electrical conductivity increased with increasing electron fluence. The improvement in these properties was attributed to irradiation-enhanced diffusion of Ti atoms, which facilitated the precipitation of Ti-rich phases from the Cu matrix. The formation of these precipitates contributed to hardening of the alloy, while the decrease in the concentration of Ti solute atoms in the Cu matrix reduced electron scattering and thereby increased the electrical conductivity. This finding challenges the conventional trade-off between mechanical strength and electrical conductivity and provides a promising strategy for designing high-strength, high-conductivity metallic materials.

More recently, the synergistic effect of pre-deformation and irradiation has attracted increasing attention. Wu et al. [28] introduced a high density of dislocations into Fe–Cu alloys through plastic pre-deformation prior to 3 MeV Fe-ion irradiation. The pre-existing dislocations acted as efficient defect sinks, promoting vacancy–interstitial recombination and suppressing irradiation-induced defect accumulation. Consequently, the pre-deformed alloy exhibited significantly improved irradiation resistance and reduced irradiation hardening compared with the undeformed counterpart, demonstrating that rational manipulation of the initial microstructure provides an effective strategy for irradiation defect engineering.

Overall, irradiation hardening results from the coupled effects of irradiation conditions, alloy chemistry, and initial microstructure. The studies discussed above illustrate the distinct contributions of accumulated dose, dose rate, irradiation-enhanced solute redistribution and precipitation, and the initial density of defect sinks. These factors influence the formation and evolution of strengthening obstacles in different ways, explaining why the magnitude of irradiation hardening varies among metallic systems and irradiation conditions. Comparisons across studies should therefore consider irradiation species and energy, dose and dose rate, temperature, alloy chemistry, and initial microstructure together rather than attributing hardening solely to accumulated dose. Understanding these coupled effects is essential for developing strategies to control irradiation hardening and improve the irradiation tolerance of metallic materials.

### 2.2. Irradiation Embrittlement

Unlike irradiation hardening, which is primarily manifested by an increase in strength and yield stress, irradiation embrittlement is characterized by the progressive degradation of fracture toughness and ductility. Although irradiation hardening and embrittlement often occur simultaneously, they are not identical degradation processes. Irradiation hardening mainly reflects the increased resistance to dislocation motion caused by irradiation-induced defect clusters [29], whereas irradiation embrittlement represents the eventual loss of plastic deformation capability and crack-arrest resistance after prolonged defect evolution [30,31]. As emphasized by Xiao et al. [12], irradiation embrittlement should be regarded as the macroscopic consequence of multiscale defect evolution rather than an independent irradiation phenomenon, because it originates from the coupled effects of point defects, defect clusters, solute redistribution, precipitate evolution and grain-boundary degradation.

From a microstructural perspective, irradiation embrittlement begins with the formation of PKAs produced by energetic neutron, ion or γ-ray irradiation. The displacement cascades generated by PKAs continuously create vacancies and SIAs, only part of which recombine immediately [32]. The remaining point defects migrate through the lattice and gradually evolve into dislocation loops, vacancy clusters and other defect complexes. These defects initially strengthen the material by increasing the resistance to dislocation glide. As irradiation-induced defects continue to accumulate and evolve, however, the ability of the material to accommodate plastic deformation may decrease, and deformation can become increasingly localized. Irradiation hardening can therefore contribute to embrittlement by restricting plastic strain accommodation and increasing the susceptibility to localized deformation and subsequent failure [12]. More importantly, the continuous accumulation of irradiation-induced point defects provides abundant diffusion pathways and defect sinks, thereby creating favorable conditions for subsequent radiation-enhanced microstructural evolution.

As irradiation proceeds, radiation-enhanced diffusion (RED) becomes increasingly important in governing defect evolution. Compared with thermal diffusion, RED significantly accelerates the migration of alloying elements through the continuous production of mobile vacancies and interstitials. Consequently, solute atoms preferentially segregate toward grain boundaries, dislocations and other defect sinks, producing the phenomenon known as radiation-induced segregation (RIS) [33,34]. RIS is widely recognized as one of the key mechanisms responsible for irradiation embrittlement because it fundamentally alters the local chemical composition at grain boundaries. In ferritic and ferritic–martensitic steels, chromium depletion accompanied by nickel- and silicon-enrichment is frequently observed near grain boundaries, weakening grain-boundary cohesion and facilitating intergranular crack initiation. Lu et al. [35] further demonstrated that irradiation-induced impurity segregation is closely associated with the ductile-to-brittle transition temperature (DBTT) shift in high-chromium ferritic/martensitic steels, providing direct evidence that grain-boundary chemistry evolution plays a decisive role in irradiation embrittlement. Besides modifying grain-boundary chemistry, RED also accelerates the redistribution of solute atoms within the matrix, providing the kinetic driving force for the nucleation and growth of irradiation-induced nanoprecipitates [36].

Besides elemental segregation, irradiation simultaneously promotes radiation-enhanced precipitation. The supersaturation of irradiation-generated point defects substantially accelerates the nucleation and growth of nanoscale precipitates that are rarely formed under conventional thermal ageing. In RPV steels, Cu-rich precipitates (CRPs) dominate embrittlement at relatively low neutron fluence, whereas Mn–Ni–Si-rich precipitates (MNSPs) become increasingly important during long-term service, particularly in modern low-Cu steels [37,38]. Wells et al. [39] showed that the composition and phase constitution of MNSPs are strongly influenced by the Ni and Mn contents of the alloy, highlighting the compositional dependence of irradiation-induced precipitation in RPV steels. These nanoscale precipitates not only act as strong obstacles to dislocation motion, further increasing irradiation hardening, but also intensify local stress concentration and facilitate microcrack nucleation. Odette and Nanstad highlighted that the evolution of irradiation-induced precipitates is one of the dominant factors governing the long-term embrittlement behaviour of RPV steels and should be explicitly incorporated into predictive embrittlement models [40]. In addition to irradiation-induced precipitation, neutron irradiation simultaneously generates transmutation gases, introducing another critical embrittlement mechanism that is particularly significant in fusion structural materials.

**Figure 2:**
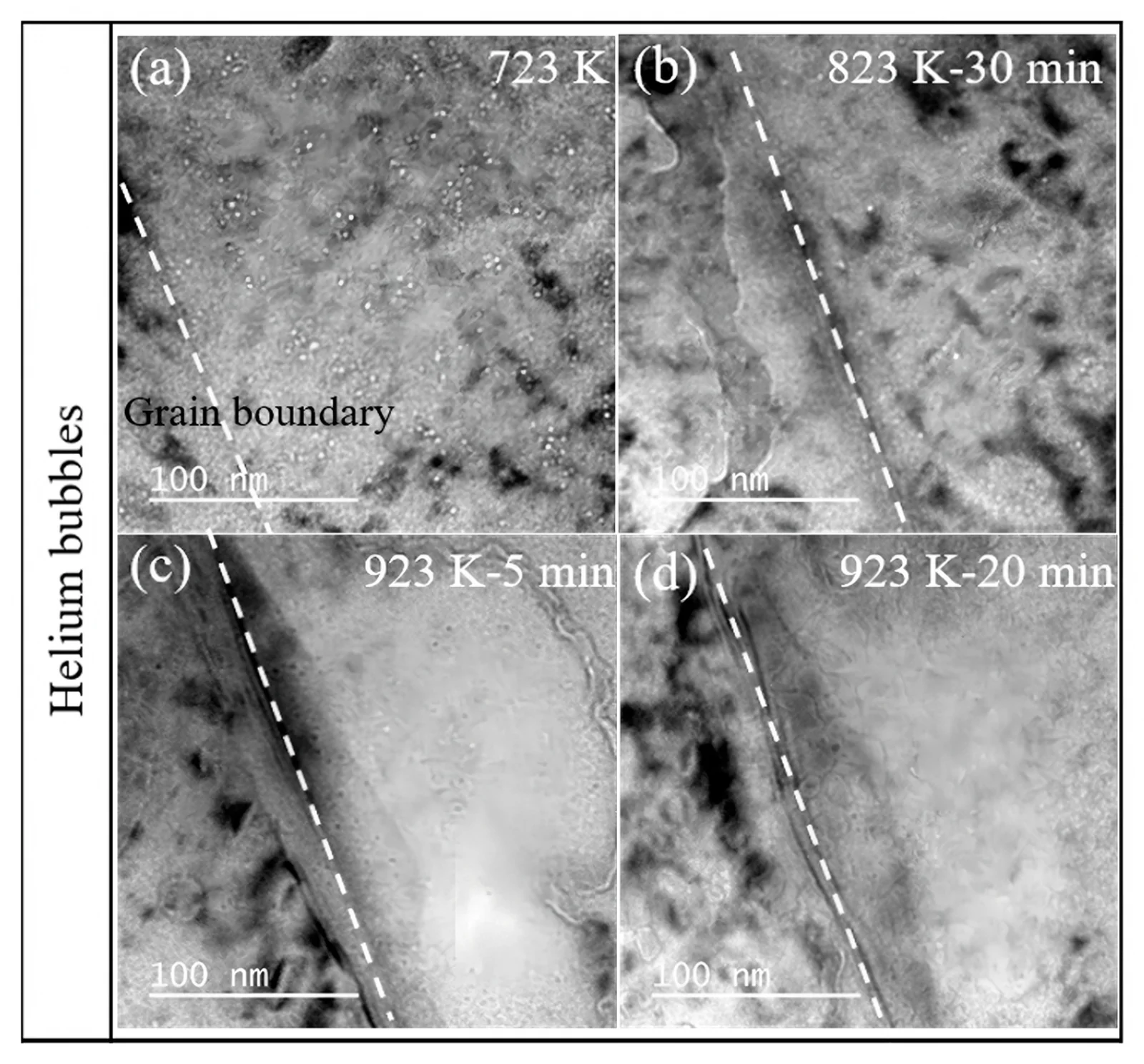
In situ TEM observations of helium bubble evolution in pre-irradiated FeCoNiCrTi_0.2_ alloy during thermal annealing: (**a**) before annealing; (**b**) after annealing at 823 K for 30 min; (**c**) after annealing at 923 K for 5 min; and (**d**) after annealing at 923 K for 20 min. The white dashed lines indicate grain boundaries. Reprinted from Ref. [42].

Another important mechanism contributing to irradiation embrittlement is the generation of transmutation gases. During fusion neutron irradiation, nuclear transmutation reactions continuously generate helium and hydrogen atoms, which can interact with irradiation-induced defects. Helium is particularly detrimental because of its extremely low solubility in metallic matrices and strong affinity for vacancies. Helium atoms are readily trapped by vacancies and vacancy clusters, promoting the formation of He–vacancy complexes and the subsequent nucleation of nanoscale helium bubbles [41]. With continued irradiation and subsequent thermal evolution, these bubbles can grow and, under appropriate conditions, migrate and coalesce into larger bubbles. He et al. [42] directly observed by in situ TEM that helium bubbles in pre-irradiated FeCoNiCrTi_0.2_ alloys tended to migrate toward grain boundaries during thermal annealing, accompanied by an increase in bubble size and a decrease in bubble density (Figure 2). The accumulation and growth of helium bubbles at grain boundaries can reduce grain-boundary fracture resistance and facilitate intergranular fracture [43]. This mechanism is particularly important in fusion reactor structural materials subjected to high neutron fluence, where helium-assisted grain-boundary embrittlement represents a major challenge to long-term structural integrity [44].

Collectively, irradiation embrittlement is therefore not controlled by a single microstructural process but by the synergistic evolution of defect accumulation, RED, RIS, irradiation-enhanced precipitation and transmutation-gas bubble formation. These mechanisms do not operate independently but evolve sequentially and interact throughout the irradiation process. Defect accumulation provides the structural basis for RED drives both elemental segregation and nanoprecipitate formation, while transmutation-gas bubbles further accelerate grain-boundary degradation under high-dose irradiation. These mechanisms continuously reduce grain-boundary cohesion, suppress plastic deformation and promote crack initiation and propagation, ultimately leading to the degradation of fracture toughness observed in irradiated metallic materials. With continued irradiation, the accumulation and coalescence of vacancy clusters eventually induce irreversible volumetric expansion, and the dominant irradiation damage gradually transitions from fracture degradation to irradiation swelling, which is discussed in the following section.

### 2.3. Irradiation Swelling

Unlike irradiation embrittlement, which primarily results from the degradation of fracture resistance and plastic deformation capability, irradiation swelling originates from the progressive accumulation of excess vacancies generated under sustained irradiation. As discussed in the previous section, displacement cascades continuously produce vacancies and SIAs, only a fraction of which recombine immediately. The surviving defects subsequently migrate toward dislocations, grain boundaries, phase interfaces and pre-existing defect clusters. When the production and survival of vacancies exceed their annihilation through recombination or absorption at defect sinks, a vacancy supersaturation is established, providing the fundamental driving force for void nucleation and volumetric swelling [45,46,47]. The classical rate-theory framework developed by Brailsford and Bullough demonstrated that swelling is governed by the competing fluxes of vacancies and interstitials toward dislocations, loops, precipitates and voids, thereby linking microscopic defect transport to the macroscopic volume-swelling rate [48].

From a microscopic perspective, the initiation of irradiation swelling is closely related to the preferential absorption of SIAs by dislocations and other biased sinks. Because dislocations generally exhibit a stronger capture efficiency for interstitials than for vacancies, the remaining matrix gradually becomes supersaturated with vacancies. These excess vacancies first aggregate into small vacancy clusters. When a cluster exceeds a critical size and becomes thermodynamically stable, it can act as a nucleus for a void. Continued vacancy absorption subsequently drives void growth, while interactions among neighbouring cavities may further promote coalescence and the formation of larger voids. Therefore, the principal evolution pathway of irradiation swelling can be summarized as point-defect generation, biased defect absorption, vacancy supersaturation, vacancy clustering, void nucleation, void growth and, ultimately, macroscopic volumetric expansion [46,49].

**Figure 3:**
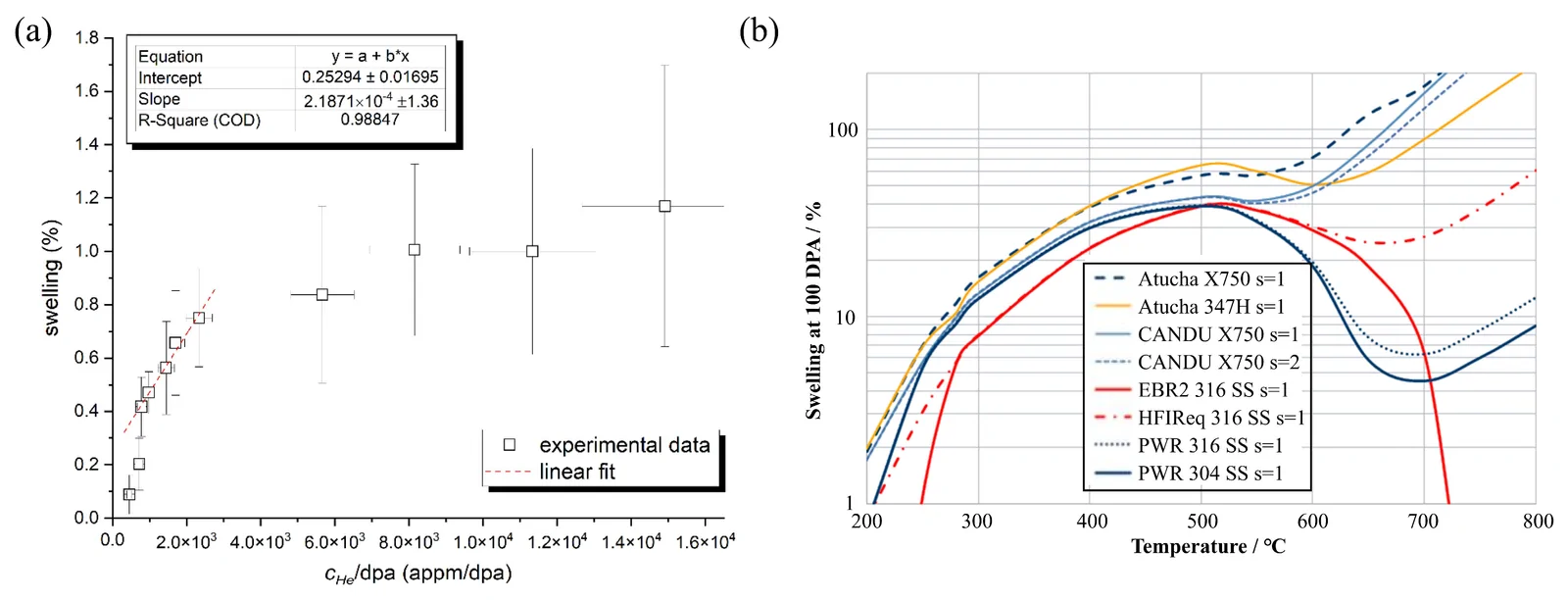
Effects of helium and temperature on irradiation swelling. (**a**) Bubble swelling in Eurofer 97 as a function of He/dpa ratio. Adapted from Ref. [52] under the CC BY license; (**b**) Calculated swelling at 100 dpa as a function of temperature for different alloys and reactor irradiation conditions. Reprinted from Ref. [51].

The evolution of irradiation-induced cavities is not governed solely by vacancy supersaturation but is also strongly influenced by irradiation-produced helium. Owing to its extremely low solubility in most metallic matrices, helium readily interacts with irradiation-induced vacancies and defect clusters, promoting the formation of He–vacancy complexes. Helium trapping can stabilize vacancies and vacancy clusters, thereby facilitating subsequent cavity nucleation [50,51]. With the continued accumulation of helium and vacancies, these clusters can develop into pressurized helium bubbles, which subsequently grow through the absorption of additional helium atoms and vacancies and may eventually evolve into voids. In helium-implanted Eurofer 97 under conditions characterized by high He/dpa ratios, an almost negligible swelling incubation period was experimentally observed, followed by bubble swelling that eventually approached saturation at higher He/dpa ratios [52] (Figure 3a). Nevertheless, helium bubbles and voids should be distinguished as different forms of irradiation-induced cavities: bubbles contain a high concentration of helium and exhibit substantial internal gas pressure, whereas voids contain large numbers of vacancies and have an internal pressure well below the equilibrium pressure.

The development of irradiation swelling is strongly dependent on temperature because defect mobility, recombination and sink absorption are all thermally activated [53]. At relatively low temperatures, vacancies possess insufficient mobility to form and grow stable voids, and irradiation damage is therefore dominated by point defects and small defect clusters. At intermediate temperatures, vacancies become sufficiently mobile to migrate and aggregate, while defect annihilation at sinks remains incomplete; under these conditions, void nucleation and growth are strongly promoted. At still higher temperatures, enhanced point-defect diffusion to grain boundaries, free surfaces and other sinks may reduce vacancy supersaturation, while thermal vacancy emission from cavities may also become increasingly important. Irradiation swelling therefore generally exhibits a characteristic intermediate-temperature window rather than increasing monotonically with temperature [54]. The position and extent of this swelling regime can, however, be further modified by helium production. Rate-theory calculations by Griffiths et al. [51] showed that the temperature dependence of swelling varies with the He/DPA ratio; under irradiation conditions with relatively high helium generation rates, helium can stabilize cavities that would otherwise tend to shrink at elevated temperatures, thereby maintaining appreciable swelling over a broader and higher temperature range (Figure 3b). These results indicate that the characteristic swelling window is governed not only by thermally activated point-defect transport but also by the coupled effects of helium production and cavity stability.

Irradiation dose is another critical variable controlling swelling evolution [53]. During the initial transient stage, a substantial fraction of irradiation-induced point defects is consumed by recombination, dislocation-loop formation and absorption at pre-existing sinks; consequently, the measured swelling remains relatively low [55]. As irradiation continues, stable void nuclei gradually develop and the cavity population enters a growth-dominated regime. Once the sink strengths and defect fluxes approach a quasi-steady state, swelling may increase approximately linearly with accumulated dose over a certain range. In their broader assessment of nuclear structural materials, Zinkle and Was identified void swelling as one of the principal bulk radiation-degradation processes in advanced fission and fusion systems, alongside irradiation hardening, embrittlement, phase instability and irradiation creep [56]. In addition to irradiation temperature and dose, the intrinsic microstructure of the material strongly determines its swelling resistance [57]. Grain boundaries, heterophase interfaces, precipitates and dispersed nanoparticles can function as defect sinks, promoting vacancy–interstitial recombination and reducing the net vacancy supersaturation available for void formation. Nanocrystalline metals and multilayered or heterogeneous materials therefore frequently exhibit delayed cavity nucleation at relatively low and intermediate doses. However, their swelling resistance should not be attributed simply to a high grain-boundary density, because grain boundaries may lose sink efficiency under prolonged irradiation, undergo structural evolution or themselves become preferential sites for helium and vacancy accumulation. The beneficial effect of defect-sink engineering consequently depends on interface stability, sink strength, irradiation temperature and accumulated dose.

Composition engineering provides another route for suppressing void growth. A representative example was reported by Yang et al. [58], who investigated irradiation-temperature-dependent swelling in equiatomic NiCoFeCrMn and NiCoFeCrPd high-entropy alloys using ion irradiation and TEM. Voids were observed in both alloys, and their swelling increased by approximately one order of magnitude as the irradiation temperature increased from 420 to 580 °C. Nevertheless, Pd alloying produced a stronger suppression of void and dislocation-loop growth than Mn alloying, which was attributed in part to localized lattice distortion and an increased defect-migration barrier. This result indicates that the irradiation tolerance of high-entropy alloys is strongly composition- and temperature-dependent and should not be generalized solely on the basis of the commonly invoked sluggish-diffusion concept.

Direct experimental evidence for dose-dependent void evolution has also been provided in ferritic/martensitic steels. Kim et al. [59] irradiated conventional and composition-engineered HT9 alloys with 3.5 MeV Fe ions to doses of up to 600 peak dpa at 450 °C and systematically characterized their void evolution. The conventional HT9 alloys displayed broadly similar swelling behaviour, whereas modification of the nitrogen content produced pronounced differences in cavity development. In particular, the high-N alloy exhibited substantially lower swelling than the low-N alloy, demonstrating that relatively subtle compositional changes can modify defect–solute interactions, precipitate evolution and the resulting resistance to void swelling.

Overall, irradiation swelling represents the cumulative outcome of long-term point-defect imbalance and cavity evolution. Its development begins with displacement-cascade-induced Frenkel-pair production, followed by biased interstitial absorption, vacancy supersaturation, vacancy clustering, stable cavity nucleation and continued void growth. Helium can facilitate this process by stabilizing vacancy clusters, whereas irradiation temperature, accumulated dose, irradiation species and energy, alloy chemistry, and defect-sink density jointly influence the incubation period and subsequent swelling rate. In particular, the effects of irradiation species and energy should be considered together with helium production conditions, as differences in damage distributions and transmutation-gas production can complicate comparisons between irradiation studies. Unlike irradiation hardening and embrittlement, which primarily degrade strength, ductility and fracture resistance, irradiation swelling directly produces irreversible dimensional changes and may compromise component clearances, geom ric tolerances and structural integrity. Under elevated temperature and applied stress, the same irradiation-induced point-defect fluxes responsible for swelling may also become directionally biased by the stress field, thereby producing time-dependent deformation known as irradiation creep, which is discussed in the following section.

### 2.4. Irradiation Creep

Unlike irradiation swelling, which is characterized by nearly isotropic volumetric expansion resulting from vacancy accumulation, irradiation creep refers to the directional, time-dependent deformation of a material subjected simultaneously to irradiation and sustained mechanical stress. It can occur at temperatures where conventional thermal creep is negligible because irradiation continuously generates nonequilibrium point defects that enhance dislocation motion and mass transport. Consequently, irradiation creep has become one of the principal deformation modes limiting the dimensional stability of structural components in fission and fusion reactors, particularly for zirconium alloys, austenitic stainless steels and ferritic/martensitic steels. The recent review by Onimus et al. [60] provides a comprehensive overview of irradiation creep in these materials and emphasizes that irradiation creep is fundamentally a defect-assisted viscoplastic deformation process rather than a simple extension of thermal creep. Similar to irradiation swelling, irradiation creep originates from the continuous production of vacancies and SIAs during displacement cascades [61]. However, under an applied stress field, the migration and absorption of these point defects become anisotropic. Instead of contributing solely to isotropic cavity growth, the defect flux preferentially promotes dislocation climb and subsequent glide along favorable crystallographic orientations, producing a permanent macroscopic strain aligned with the applied stress [61]. Therefore, irradiation creep can be regarded as the combined consequence of irradiation-generated point defects and stress-induced asymmetry in defect transport.

Among the numerous mechanisms proposed over the past several decades, stress-induced preferential absorption (SIPA) remains one of the most influential theoretical frameworks. In this mechanism, irradiation continuously supplies vacancies and SIAs, while the applied stress modifies the interaction energy between point defects and dislocations [62]. Consequently, dislocations with different Burgers-vector orientations absorb unequal numbers of vacancies and interstitials, resulting in preferential dislocation climb and the accumulation of directional plastic strain. The SIPA mechanism therefore transforms an initially isotropic defect flux into anisotropic deformation under stress. Detailed descriptions of SIPA and its historical development are provided in the reviews by Onimus et al. [60] and Adamson et al. [63]. Although SIPA successfully explains many experimental observations, it is no longer regarded as the sole mechanism governing irradiation creep. Recent reviews have emphasized that the relative contribution of SIPA depends strongly on alloy composition, irradiation temperature, dose, initial dislocation density and microstructural evolution during irradiation [64]. In particular, irradiation creep in recrystallized zirconium alloys and ferritic steels often cannot be fully interpreted using the classical SIPA framework alone [63].

Another widely accepted mechanism is the climb-enhanced dislocation glide (CEG), also referred to as stress-induced climb and glide (SICG). Unlike SIPA, which attributes deformation primarily to anisotropic point-defect absorption by dislocations, this mechanism emphasizes the synergistic interaction between irradiation-enhanced dislocation climb and stress-driven glide [65]. Under irradiation, the continuous absorption of vacancies and SIAs enables dislocations to climb around irradiation-induced obstacles, including defect clusters, dislocation loops and nanoscale precipitates. Once these obstacles are bypassed, the applied stress drives further dislocation glide, producing a sustained creep strain. Because the strain is generated mainly by glide while the rate is controlled by climb, this mechanism is often referred to as a climb-controlled glide process. For cold-worked zirconium alloys, climb-enhanced glides have been considered an important irradiation-creep mechanism, particularly because it can account for aspects of creep anisotropy that are not fully reproduced by the classical SIPA model alone [63]. The irradiation creep rate is influenced by multiple irradiation and material parameters [66]. Applied stress determines the driving force for directional deformation, whereas irradiation temperature governs point-defect mobility and dislocation climb kinetics. At relatively low temperatures, limited defect mobility suppresses climb processes, resulting in low creep rates. Increasing the irradiation temperature promotes defect diffusion and accelerates climb-assisted deformation. However, at sufficiently high temperatures, thermal creep becomes increasingly important, making it difficult to distinguish irradiation creep from conventional high-temperature creep. Irradiation dose and dose rate also play essential roles because they determine the steady-state concentration of irradiation-induced point defects. In addition, alloy composition, grain size, dislocation density and defect-sink distribution collectively influence the partitioning of point defects and therefore modify irradiation-creep behavior [63].

**Figure 4:**
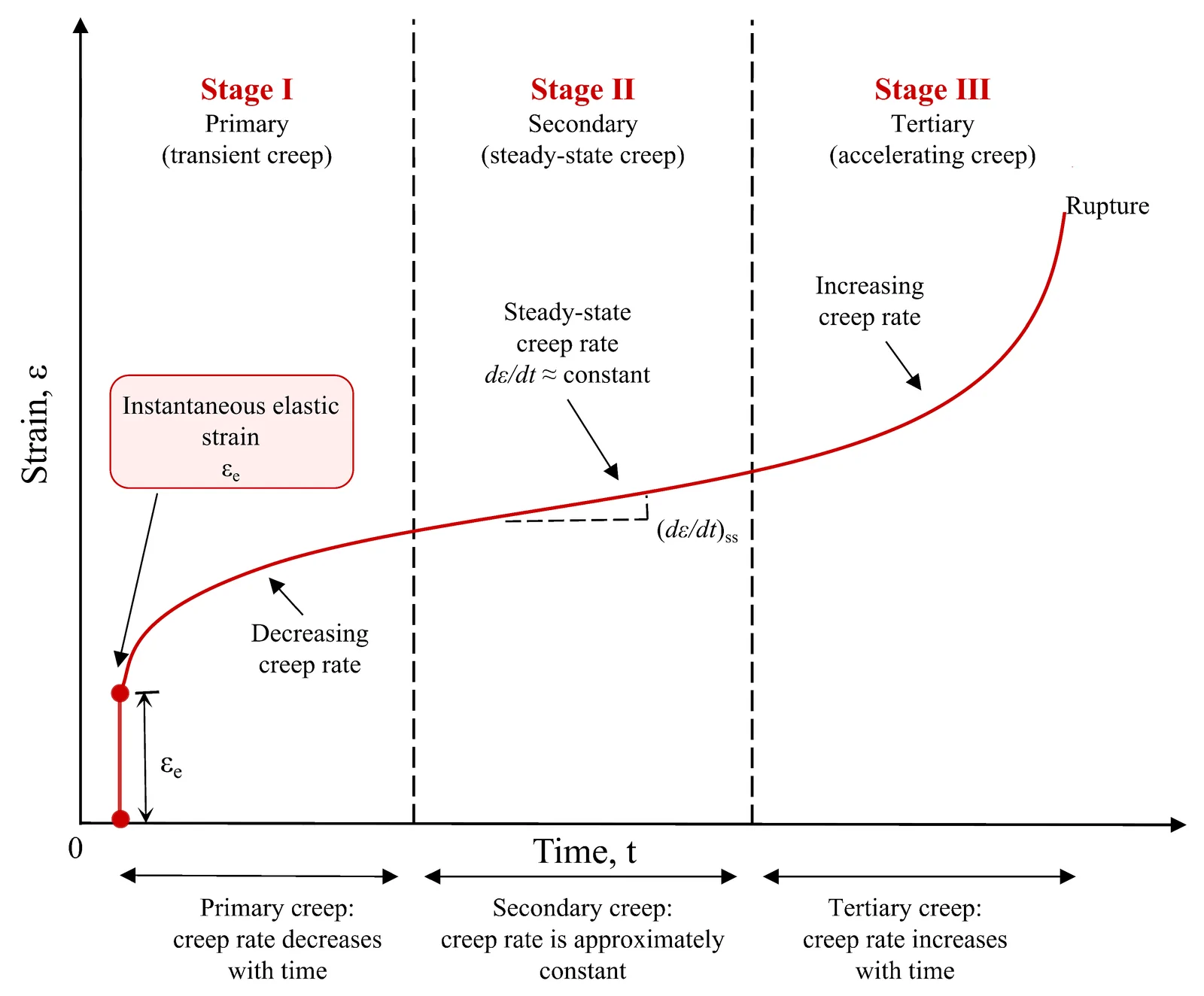
Schematic illustration of the typical creep strain evolution under irradiation, showing the primary, secondary and tertiary creep stages.

Representative experimental evidence has been obtained from zirconium alloys used in nuclear fuel cladding. Adamson et al. [63] systematically summarized more than five decades of in-reactor observations and demonstrated that irradiation creep exhibits an approximately linear dependence on applied stress under normal reactor operating conditions while remaining strongly affected by neutron fluence, irradiation temperature and crystallographic texture (Figure 4). Their review also shows that creep behavior varies considerably between recrystallized and cold-worked zirconium alloys because of differences in the initial dislocation density and dominant deformation mechanisms.

Overall, irradiation creep represents the directional manifestation of irradiation-induced point-defect transport under an applied stress field. Unlike irradiation swelling, which mainly reflects isotropic vacancy accumulation, irradiation creep originates from the stress-assisted redistribution of irradiation-generated defects through mechanisms such as SIPA and climb-enhanced dislocation motion. Although the relative importance of these mechanisms varies among different alloy systems, they all demonstrate that irradiation-induced point defects not only degrade structural integrity but also fundamentally alter the kinetics of dislocation motion. Together with irradiation hardening, embrittlement and swelling, irradiation creep completes the principal framework of irradiation-induced degradation in metallic materials.

## 3. Functional Utilization of Irradiation-Induced Defects in Metallic Materials

Although irradiation-induced defects have traditionally been regarded as a primary cause of radiation damage in metallic materials, increasing evidence suggests that irradiation can also be deliberately employed to regulate microstructures and material properties under appropriate conditions. Whether irradiation causes material degradation or enables beneficial property regulation depends on the irradiation conditions and the resulting defect evolution and microstructural changes. By controlling defect generation, migration, interaction, and annihilation, irradiation can be exploited to tailor the structural and functional characteristics of metallic materials. However, the resulting property changes may also involve irradiation-induced compositional redistribution, phase transformation, grain refinement, and surface melting or solidification, depending on the irradiation conditions and material system. Accordingly, the functional utilization discussed in this section encompasses both defect-mediated property regulation and irradiation-assisted microstructural modification, without assuming that the observed improvements originate exclusively from irradiation-induced defects. This section reviews these developments, focusing on structural modification and mechanical-property enhancement, irradiation-regulated magnetic properties, and surface/interface functionalization.

### 3.1. Structural Modification and Mechanical Property Enhancement

Traditionally, irradiation has been regarded as a degradation process because irradiation-induced defects usually lead to hardening, embrittlement and dimensional instability. However, recent studies have demonstrated that under controlled irradiation conditions these defects can also be deliberately utilized as effective microstructural engineering tools [67]. High-energy particle irradiation can promote grain refinement, accelerate recrystallization, regulate phase distribution and modify surface morphology, thereby significantly improving hardness, wear resistance and strength. Consequently, irradiation is increasingly recognized not only as a source of radiation damage but also as a promising approach for structural modification and mechanical-property enhancement.

Hydrogen-ion irradiation has recently been demonstrated as an effective approach for regulating the crystallization behavior of Fe_86_B_14_ metallic glass. Compared with the unirradiated alloy, irradiation introduced numerous defect-assisted nucleation sites that promoted the formation of finer and more uniformly distributed nanocrystals during subsequent annealing, thereby improving the soft magnetic properties. The study indicates that irradiation-induced defects can be intentionally utilized to manipulate crystallization kinetics rather than merely causing structural degradation. Similar defect-assisted crystallization behavior has also been observed in lunar glasses exposed to simulated solar-wind irradiation. Hydrogen implantation produced abundant Fe-rich clusters that subsequently acted as preferential heterogeneous nucleation sites during thermal treatment, leading to uniform nanocrystalline structures. This phenomenon provides an important insight into the utilization of irradiation for tailoring extraterrestrial materials under space environments [68].

**Figure 5:**
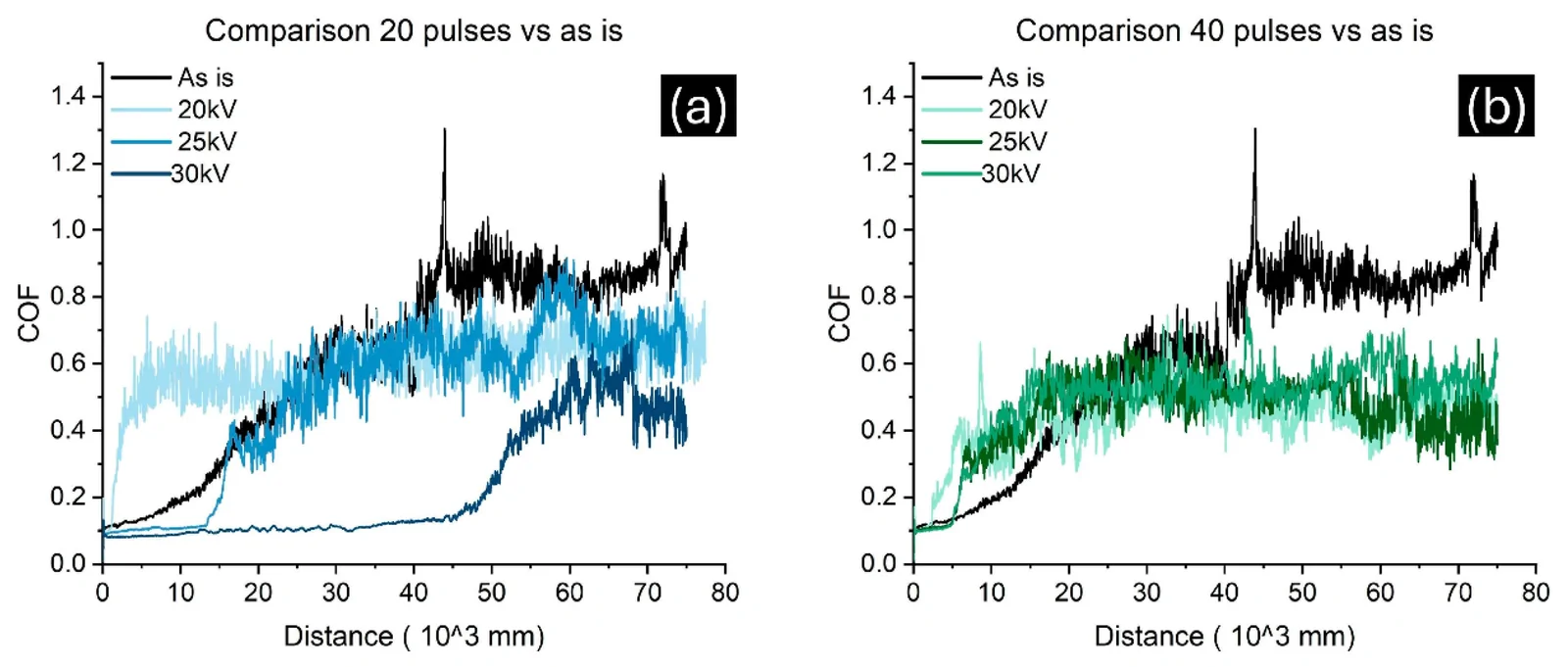
Evolution of friction coefficient during wear tests of LEHCEB-treated 17-4PH stainless steel after (**a**) 20 pulses and (**b**) 40 pulses, compared with the as-sintered condition. Reprinted from Ref. [69].

Besides bulk nanocrystallization, irradiation can also be employed for localized surface modification. Owing to the extremely high heating and cooling rates during low-energy high-current electron beam (LEHCEB) irradiation, rapid melting and solidification occur within the near-surface layer, producing refined grains, homogeneous phase distributions and residual compressive stresses. These microstructural changes substantially improve hardness, wear resistance and crack resistance without altering the bulk microstructure. Ivanov et al. [67] demonstrated that LEHCEB irradiation refined the Ni_3_Al matrix grains and TiC particles in Ni_3_Al–TiC composites. For the Ni_3_Al–30 vol% TiC composite, irradiation at an energy density of 18 J cm^−2^ increased the TiC volume fraction in the near-surface layer from 30.7% to 44.9% and enhanced the microhardness by approximately 50%. Similar LEHCEB-based surface engineering has also been applied to metallic alloys. Fabiocchi et al. [69] investigated the effect of LEHCEB treatment on 17-4PH stainless steel produced via binder jetting and found that electron beam irradiation induced significant modifications in the near-surface microstructure and improved the tribological performance of the treated alloy (Figure 5). These results further demonstrate that irradiation-assisted surface modification enables localized regulation of near-surface microstructures while maintaining the overall characteristics of metallic materials.

Collectively, these studies demonstrate that irradiation-induced defects can serve as effective microstructural engineering tools rather than merely acting as sources of radiation damage. Through controlled defect generation, grain refinement, rapid surface remelting and phase redistribution, irradiation enables simultaneous improvements in hardness, strength and wear resistance. These findings establish the conceptual basis for utilizing irradiation as a versatile approach for structural modification and mechanical-property enhancement.

### 3.2. Irradiation-Regulated Magnetic Properties

Besides modifying the mechanical behavior of metallic materials, irradiation-induced defects can also be deliberately utilized to regulate magnetic properties. Unlike conventional alloying, irradiation enables post-processing of magnetic materials without necessarily changing their bulk alloy composition. Point defects, local lattice distortions and irradiation-induced damage regions can influence magnetic exchange interactions and domain-wall motion, thereby providing an effective route for tailoring coercivity and magnetic ordering [13]. Consequently, irradiation defect engineering has become an attractive strategy for functional magnetic-material design.

A representative example was reported by Dempsey et al. [70], who irradiated Sm_2_Fe_17_N_3_ permanent-magnet particles with 5-GeV Pb^56+^ ions. At the highest fluence of 2 × 10^13^ ions cm^−2^, the coercivity increased fivefold from 0.3 to 1.5 T at 5 K. Mössbauer spectroscopy revealed irradiation-damaged regions, while magnetization measurements showed characteristic domain-wall pinning behavior. Orientation-dependent magnetic measurements further indicated that the pinning defects were anisotropic in shape. Based on a strong-pinning model, the authors attributed the enhanced coercivity to elongated, discontinuous irradiation-induced damage regions that impede domain-wall motion, with an estimated defect radius of approximately 2 nm. This study demonstrates that irradiation-induced defects can improve resistance to magnetization reversal by introducing effective domain-wall pinning centers.

Beyond enhancing coercivity, irradiation-induced defects can also regulate magnetic ordering. Eggert et al. [71] investigated initially antiferromagnetic B2-ordered FeRh thin films irradiated with 25-keV Ne^+^ ions. Irradiation increased the low-temperature magnetization and shifted the antiferromagnetic-to-ferromagnetic transition toward lower temperatures. Conversion electron Mössbauer spectroscopy revealed an increasing ferromagnetic contribution at room temperature with increasing ion fluence. Positron annihilation spectroscopy confirmed an increase in open-volume defect concentration, while extended X-ray absorption fine-structure analysis indicated enhanced static structural disorder without substantial local Fe–Rh intermixing. The authors proposed that irradiation-induced defects and lattice distortions break local structural symmetry and modify the electronic environment, thereby promoting ferromagnetic ordering. These findings demonstrate the potential of irradiation-induced structural disorder to stabilize ferromagnetic behavior in a material that is predominantly antiferromagnetic at room temperature before irradiation.

Collectively, these studies demonstrate that irradiation-induced defects are not merely detrimental microstructural imperfections but can also serve as effective regulators of magnetic behavior. By controlling irradiation conditions, such as ion species and fluence, defects and local structural disorder can be introduced to enhance coercivity through domain-wall pinning or promote ferromagnetic ordering through changes in the local structural environment. These findings broaden the application scope of irradiation beyond conventional radiation-damage studies, highlighting its potential as a defect-engineering strategy for functional magnetic-material design.

### 3.3. Irradiation-Enabled Surface and Interface Functionalization

In addition to modifying bulk microstructures and magnetic properties, energetic-particle irradiation can be used to selectively engineer the near-surface region of metallic materials. Because ions and low-energy electron beams deposit most of their energy within a limited depth, irradiation can introduce nonequilibrium defects, alloying elements, amorphous layers, nanocrystalline phases and residual stresses without substantially changing the bulk composition or geometry of the component. The resulting surface layer may exhibit markedly different chemical, electrochemical, tribological and biological properties from the untreated substrate. Low-energy high-current electron-beam processing, ion implantation and plasma immersion ion implantation are therefore increasingly employed as localized surface-functionalization techniques for improving corrosion resistance, wear resistance and biological performance. Reviews of low-energy high-current electron-beam processing have shown that rapid surface melting and solidification can produce graded, metastable and refined surface structures in steels, aluminium alloys, titanium alloys and hard metals [14,15]. The principal advantage of irradiation-enabled surface engineering is that the modified layer is metallurgically integrated with the substrate rather than deposited as a mechanically bonded coating. During ion implantation, energetic ions penetrate the substrate and generate collision cascades, resulting in atomic mixing, defect production and compositional gradients. Depending on the ion species and processing conditions, implantation may promote surface amorphization, nanocrystalline-phase formation or the development of compact oxide, nitride or carbide-containing layers. In electron-beam processing, intense pulsed heating can melt a thin surface layer within microseconds, followed by extremely rapid solidification. This process refines grains and second-phase particles, homogenizes the surface composition and may introduce beneficial compressive stresses. However, excessive energy or implantation voltage may also increase sputtering, roughness and radiation damage, indicating that surface functionalization requires careful optimization of ion energy, fluence, pulse number and treatment temperature [72,73].

**Figure 6:**
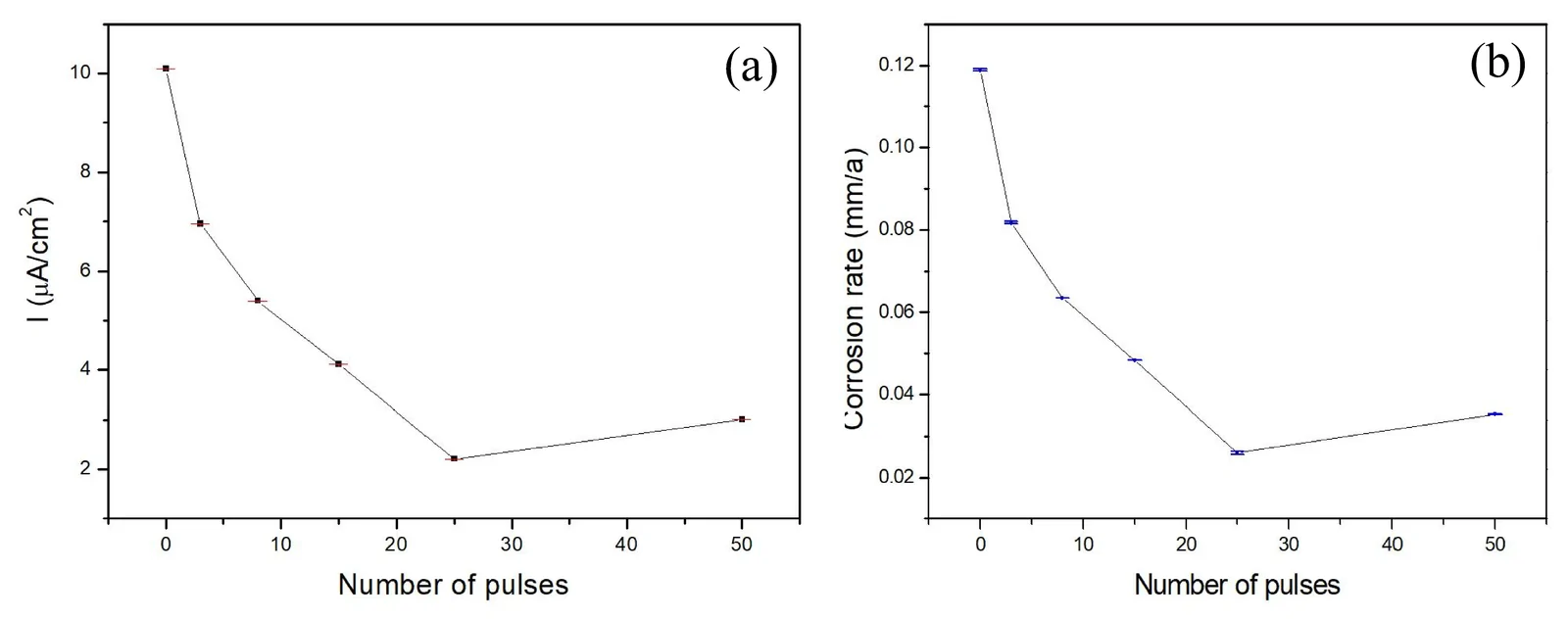
Self-corrosion current density (**a**) and corrosion rate of 40CrNiMo7 steel (**b**) before and after HCPEB irradiation. Reprinted from Ref. [74].

Corrosion resistance is one of the important functional properties investigated in irradiation-modified metallic surfaces. The enhancement generally originates from irradiation-induced regulation of surface chemistry and microstructure, including compositional redistribution, grain refinement and the formation of modified surface layers that improve corrosion resistance. For example, high-current pulsed electron beam (HCPEB) irradiation of 40CrNiMo7 steel generated a refined and compositionally homogenized surface layer, resulting in a significant decrease in corrosion current density and corrosion rate [74] (Figure 6). The improved corrosion resistance was associated with surface compositional homogenization and microstructural refinement after HCPEB treatment. Titanium ion implantation has also been used to modify the corrosion behaviour of 316L stainless steel [72]. In the untreated alloy, second-phase inclusions and heterogeneous regions provided preferential sites for pitting and intergranular corrosion. After Ti implantation, the outermost surface became more homogeneous and substantially amorphized, suppressing localized corrosion. Nevertheless, the study also found that excessively high implantation voltages increased surface roughness and irradiation damage, thereby reducing part of the corrosion benefit. This result is particularly important because it demonstrates that irradiation intensity does not necessarily correlate monotonically with functional improvement. Plasma-based nitrogen implantation offers a related route for achieving simultaneous corrosion and wear improvement in austenitic stainless steels [75]. Low-temperature nitrogen implantation can form nitrogen-supersaturated expanded austenite, frequently denoted as γN, without producing the extensive chromium-nitride precipitation associated with conventional high-temperature nitriding. This modified phase increases surface hardness and wear resistance while preserving or improving corrosion performance under suitable implantation conditions. Such coupled functional enhancement was demonstrated in 1Cr18Ni9Ti austenitic stainless steel treated using plasma-based low-energy ion implantation.

Irradiation-assisted surface functionalization is particularly attractive for biodegradable magnesium alloys because their rapid corrosion in physiological media remains a major barrier to biomedical application. Plasma immersion ion implantation can introduce corrosion-resistant elements or functional groups while retaining the favourable bulk mechanical properties of the Mg substrate [76]. In AZ91 magnesium alloy, plasma implantation and deposition of Al, Zr or Ti generated a multilayered surface consisting of implanted metals, their oxides and magnesium oxides. Among the investigated elements, Al treatment produced the clearest positive shift in open-circuit potential and the strongest improvement in corrosion resistance in artificial physiological fluids. Nitrogen and carbon-containing plasmas have similarly been employed to improve magnesium surfaces. Nitrogen plasma implantation enhanced corrosion resistance when the implantation energy and treatment duration were appropriately selected, although excessive sputtering and possible nitride formation could become detrimental [77]. Carbon dioxide plasma implantation into AZ31 magnesium alloy generated a carbon- and oxygen-containing surface layer and improved electrochemical performance in physiological environments. More sophisticated implantation strategies can combine corrosion protection with explicit biological functionalization. Carboxyl-ion implantation into ZK60 magnesium alloy grafted carboxyl-containing species onto the surface, reduced the degradation rate and improved in vitro biocompatibility. Unlike conventional metallic-ion implantation, this approach introduced a chemically active organic functional group, illustrating that irradiation can be used not only to strengthen a passive layer but also to tailor biological interactions at the metal–tissue interface [78]. Hafnium implantation into WE43 magnesium alloy provides another representative example [79]. The implanted layer consisted predominantly of Hf-containing oxides and MgO, which retarded degradation in simulated body fluid and simultaneously improved cell attachment and cytocompatibility. This combination of corrosion control and biological response demonstrates that implanted surface layers can be designed as multifunctional interfaces rather than merely protective barriers. Overall, the electrochemical results demonstrate changes in corrosion behavior; however, because surface-composition changes, the formation of oxide- or compound-containing layers, and surface-structural modifications may occur simultaneously, their individual contributions are not always distinguishable from the reported evidence.

Wear and friction constitute another mature application of irradiation-based surface engineering. Nitrogen implantation into steel has long been shown to improve wear resistance by increasing near-surface hardness and modifying the defect and phase structure. In two tool steels implanted with 50keV nitrogen ions, wear resistance increased with implantation dose over the investigated range, while measurable hardening emerged at the higher doses [80]. Low-energy high-current electron-beam irradiation provides a more intense route for producing wear-resistant surface layers through rapid melting, refinement and resolidification. The physical basis of this method was established in early studies showing that its strongest structural changes occur in the rapidly quenched near-surface region [67]. In Ni_3_ Al-TiC composites, pulsed electron-beam treatment reduced TiC particle size by approximately two orders of magnitude, increased the TiC volume fraction in the modified layer and produced an approximately 1.5-fold increase in microhardness. However, the same study reported a decrease in bending strength, indicating that improvements in surface hardness do not necessarily translate into better overall mechanical performance. This trade-off highlights the importance of evaluating irradiation-induced property changes in relation to the specific mechanical requirements of the intended application. A related surface-composite strategy involves first depositing a thin TiC powder layer on Ni_3_ Al and then using electron-beam irradiation to melt and mix the coating with the substrate. This treatment produced a near-surface composite layer approximately 4.5 μm thick, reduced surface-crack density and increased wear resistance by a factor of 5.6 relative to TiC-free irradiated Ni_3_ Al.

Surface irradiation can also impart antibacterial functionality to titanium implants. Ion implantation and ion-beam mixing permit antibacterial elements to be introduced into the outermost surface while maintaining the mechanical properties of the Ti substrate. An early systematic study compared Ca, N and F implantation, several oxide and nitride treatments, and Ar-ion-beam mixing with Ag, Sn, Zn and Pt [81]. The results demonstrated that antibacterial behaviour depends strongly on both the implanted species and the resulting surface state. The biomedical significance of such treatments has subsequently expanded beyond antibacterial activity to include osseointegration, cell adhesion and controlled ion release. Reviews of antibacterial titanium surfaces emphasize that ion implantation is attractive because it can modify near-surface chemistry without producing a thick, weakly bonded coating. However, functional performance depends on ion stability, release kinetics, toxicity and the durability of the implanted layer.

Overall, irradiation-enabled surface and interface functionalization relies on the deliberate confinement of nonequilibrium structural and chemical changes to a shallow near-surface region. Ion implantation can introduce passivating, hardening or biologically active species; plasma immersion treatments are suitable for components with complex geometries; and pulsed electron beams can induce rapid melting, phase refinement and metallurgical surface alloying. These processes can improve corrosion resistance, wear resistance and biological functionality without requiring major changes to the bulk material. Accordingly, the functional improvements observed in irradiation-treated surfaces may reflect varying contributions from irradiation-induced defect evolution, changes in surface composition and phase constitution, and near-surface microstructural evolution, depending on the treatment conditions and material system. Nevertheless, the benefits are highly sensitive to processing parameters. Excessive implantation damage, sputtering, roughening, brittle-phase formation or insufficient modified-layer thickness may offset the desired functional gains. Future implementation therefore requires quantitative control of penetration depth, defect density, surface composition and long-term interfacial stability.

To facilitate comparison, Table 1 summarizes the irradiation conditions, microstructural changes, and resulting property changes reported in selected representative studies discussed in Section 3.1, Section 3.2 and Section 3.3.

## 4. Challenges and Future Perspectives

The rapid development of irradiation defect engineering has considerably expanded the application of irradiation technologies from conventional radiation-damage studies to functional material design. Nevertheless, several challenges remain before irradiation can be widely adopted as a reliable engineering strategy. One of the primary challenges is the limited understanding of irradiation-induced defect evolution across multiple length and time scales. In particular, how irradiation parameters and initial microstructures govern the type, density and spatial distribution of defects, and how these defect populations determine material properties, remain important open questions. Future investigations should integrate advanced characterization techniques, such as in situ TEM and APT, with multiscale simulations to establish quantitative relationships between irradiation parameters, defect evolution and material performance. Another challenge is the transferability of ion-irradiation results to neutron-irradiation conditions, as differences in damage profiles, dose rates and transmutation effects may produce distinct defect populations even at comparable damage levels. Systematic comparative studies are therefore needed to clarify the applicability and limitations of ion irradiation in simulating neutron damage.

The stability of irradiation-modified structures during subsequent thermal exposure also requires attention, as defect recovery and microstructural evolution may affect the retention of improved properties. Future studies should therefore assess both the immediate property changes after irradiation and their stability under subsequent thermal treatment or relevant service conditions. Pulsed electron-beam surface treatments, including LEHCEB and HCPEB, have shown promise for improving surface microhardness, wear resistance and corrosion resistance in different metallic systems through rapid surface melting and associated microstructural refinement or homogenization. However, further research is needed to establish suitable processing windows for beam energy density and pulse number, ensuring effective surface modification while minimizing surface cracking and detrimental residual stresses. From an application perspective, irradiation research should also place greater emphasis on the coordinated optimization of multiple material properties. While irradiation can significantly improve hardness, corrosion resistance, wear resistance and magnetic performance, enhancement of one property may be accompanied by deterioration of another. Consequently, balancing structural reliability with multifunctional performance remains a critical issue for engineering applications. In addition, combining advanced characterization, computational modeling and data-driven approaches is expected to accelerate the rational design of irradiation-tolerant and multifunctional metallic materials.

## 5. Conclusions

Metallic materials operating in irradiation environments inevitably undergo microstructural evolution driven by irradiation-induced defects, resulting in irradiation hardening, embrittlement, swelling and creep. This review systematically summarized the fundamental mechanisms responsible for these radiation damage phenomena, highlighting the generation, migration and interaction of irradiation-induced defects and their influence on the structural stability and mechanical performance of metallic materials. Beyond conventional radiation damage, increasing attention has been devoted to the functional utilization of irradiation-induced defects. Recent studies demonstrate that controlled irradiation can effectively regulate microstructures, enhance mechanical properties, tailor magnetic behavior, and improve surface-related properties such as corrosion resistance, wear resistance and biocompatibility. These advances indicate that irradiation is gradually evolving from a source of material degradation into an effective tool for defect engineering and functional material design. Overall, this review highlights the transition of irradiation research from understanding radiation damage to exploiting irradiation-induced defects for material design, providing a useful reference for the development of next-generation multifunctional metallic materials.

## Figures and Tables

**Table 1:** Comparison of irradiation conditions, microstructural changes, and resulting property changes in representative metallic materials.

Material	Irradiation Type	Energy/Energy Density	Fluence/Dose or Pulses	Irradiation Temperature	Main Microstructural Changes	Resulting Property Changes
Fe_86_B_14_ [68]	H^+^ irradiation	60 keV	~1 × 10^17^ ions cm^−2^	Not reported	Structural fluctuations and clusters formed in the irradiated surface layer (~10 nm); after annealing at 653 K for 5 s, finer nanocrystals (5–8 nm) formed near the surface	Relative permeability at 10 kHz increased from 1308 to 1442 (~10.2%) after irradiation and subsequent annealing
Ni_3_Al–30 vol% TiC composite [67]	LEHCEB	30 kV; 18 J cm^−2^	10 pulses	Not reported	Refinement of Ni_3_Al matrix grains and TiC particles (35 ± 9 nm); increased TiC fraction from 30.7 to 44.9 vol%; surface cracking and internal stresses	Microhardness increased to ~1.5 times the initial value, while ultimate bending strength decreased from 532 ± 146 to 440 ± 49 MPa
17-4PH Steel [69]	LEHCEB	30 kV; 4.9 J cm^−2^	20 pulses	Not reported	Surface remelting and rapid solidification; reduced surface roughness and porosity; formation of a modified layer with columnar martensitic and ferritic grains (1–3 μm wide)	Prolonged low-friction stage (COF ≈ 0.1) and improved tribological performance compared with the as-sintered condition
Sm_2_Fe_17_N_3_ [70]	Pb^56+^ heavy-ion irradiation	5 GeV	2 × 10^13^ ions cm^−2^	Not reported	Formation of anisotropic irradiation-damaged regions acting as domain-wall pinning centers	Coercivity increased fivefold from 0.3 to 1.5 T at 5 K
B2-FeRh thin films [71]	Ne^+^ irradiation	25 keV	0.125 ions nm^−2^	Not reported	Increased open-volume defect concentration, evidenced by a decrease in positron diffusion length from 19.7 to 5.68 nm	Increased low-temperature magnetization and emergence of a ferromagnetic contribution at 300 K
40CrNiMo7 Steel [74]	HCPEB	27 kV; 3 J cm^−2^	25 pulses	Not reported	Surface remelting and grain refinement; formation of columnar dendrites in the modified layer; increased austenite (γ-Fe) diffraction intensity	Corrosion current density decreased from 10.097 to 2.2099 μA cm^−2^, and corrosion rate decreased from 0.12 to 0.02 mm year^−1^
316L stainless steel [72]	Ti-ion implantation	20, 40, 60 and 80 kV	~2 × 10^17^ ions cm^−2^	Not reported	At 20 kV, a Ti-enriched layer (~100 nm) and an amorphous surface layer (~15 nm) formed. Surface roughness increased with implantation voltage, particularly at 60–80 kV.	Ti implantation improved corrosion resistance, but higher implantation voltages increased the passivation current density.
1Cr18Ni9Ti stainless steel [75]	Plasma-source low-energy N-ion implantation	−2 kV (pulsed bias)	Ion-current density: 0.44–0.63 mA cm^−2^; 4 h	380 °C	Formation of nitrogen-supersaturated expanded austenite (γ_n_); nitrogen content increased from ~26 to ~32 at.% with increasing ion-current density	Microhardness reached ~22 GPa; wear and pitting corrosion resistance improved, particularly at higher nitrogen content; general corrosion resistance remained comparable to untreated steel

## Data Availability

No new data were created or analyzed in this study. Data sharing is not applicable to this article.
